# Ideating iDNA: Lessons and limitations from leeches in legacy collections

**DOI:** 10.1371/journal.pone.0212226

**Published:** 2019-02-22

**Authors:** Mark E. Siddall, Megan Barkdull, Michael Tessler, Mercer R. Brugler, Elizabeth Borda, Evon Hekkala

**Affiliations:** 1 Division of Invertebrate Zoology, American Museum of Natural History, New York, New York, United States of America; 2 New College of Florida, Sarasota, Florida, United States of America; 3 Biological Sciences Department, NYC College of Technology, City University of New York, Brooklyn, New York, United States of America; 4 Department of Science and Math, Texas A&M University San Antonio, San Antonio, Texas, United States of America; 5 Department of Biological Sciences, Fordham University, Bronx, New York, United States of America; University of Hyogo, JAPAN

## Abstract

Indirect methods for conducting faunal inventories present great promise, and genomic inventories derived from environmental sources (eDNA) are improving. Invertebrate ingested DNA (iDNA) from terrestrial leeches in the family Haemadipsidae has shown potential for surveying vertebrates and biodiversity monitoring in protected areas. Here we present an initial, and critical, evaluation of the limitations and biases of current iDNA protocols for biodiversity monitoring using both standard and NGS barcoding approaches. Key findings include the need for taxon relevant multi-locus markers and reference databases. In particular, the limitations of available reference databases have profound potential to mislead and bias eDNA and iDNA results if not critically interpreted. Nevertheless, there is great potential for recovery of amplifiable DNA from gut contents of invertebrate museum specimens which may reveal both temporal patterns and cryptic diversity in protected areas with increased efficiency. Our analyses of ingested DNA (iDNA) from both freshly stored and previously collected (legacy) samples of terrestrial leeches successfully identified vertebrates from Myanmar, Australia and Madagascar and indicate the potential to characterize microbial communities, pathogen diversity and interactions at low cost.

## Introduction

Inventorying strategies are key to successful conservation programs. Overall species composition in protected areas helps to identify management priorities and informs the implementation of usage programs [1]. Furthermore, the success of a particular conservation effort [2] relies on effective longitudinal biodiversity monitoring in ways that also allow for evaluation of environmental change, ecosystem health, and the emergence of pathogens [3, 4].

Live trapping, line transects, and track surveys are common inventory methods for terrestrial tetrapods, each with its own inherent limitations and biases [5, 6]. Passive camera trapping is often considered more efficient especially in terms of human resources and expertise [7, 8]. Insofar as the likelihood of a species triggering a sensor is inversely proportional to body size [9], camera trapping the majority of mammals requires specific modifications [10] or the use of intermittent video capture instead [11]. Meanwhile reptiles and amphibians, which are more imperiled than mammals [12, 13], are represented by comparatively few camera trap studies [14]. Of the foregoing, only live-trapping and active capture allow for comprehensive genetic biodiversity monitoring of the fullest range of vertebrate diversity in protected areas while allowing for demographic studies and the detection of cryptic species [3, 15, 16].

In aquatic habitats, passive environmental DNA (eDNA) sampling has proven robust when employed in a longitudinal design [17] and useful in detecting invasive and rare aquatic species [18, 19]. Though eDNA is ill-suited to terrestrial monitoring, a burgeoning use of an invertebrate ingested DNA (iDNA) approach shows promise [20, 21, 22, 23]

Sampling iDNA from leeches encompasses some of the benefits of other survey strategies [24] for those leech groups that have both sanguivorous and terrestrial life-history strategies [25]. Much like investigators in track and transect surveys, leeches are actively sampling their environment, but unlike biting flies leeches do not travel large distances between blood meals [26]. Residual mitochondrial DNA remains detectable in the gut of leeches for months post-feeding [27] and it is becoming clear that terrestrial leeches are particularly adept at revealing small animal diversity in ways otherwise only possible with trapping and active collecting [26].

Each of cytochrome b, 12S rDNA, 16S rDNA and cytochrome *c* oxidase subunit 2 gene have been explored as a barcode locus in iDNA isolates from mosquitoes, carrion flies, biting midges, ticks and leeches [21, 27, 28, 29, 30]. The mitochondrial ribosomal genes are among the more taxonomically universal markers [31], though rarely are they used in combination. Here we explore several iDNA strategies toward the recovery iDNA in recent as well as >10 year old museum collections of terrestrial leeches while also assessing leech gut associated bacterial symbionts and vertebrate trypanosome parasites detectable in blood meals.

## Materials and methods

### Conventional sequencing

In order to directly compare the utility of short 12S rDNA fragments to published 16S rDNA sequences in the identification of vertebrate iDNA, we leveraged the same samples that were collected and identified to species, and served as template, in previous studies [26, 32]. Briefly, 761 individual leeches were collected in Yunnan and Hainan provinces in China (in 2014), from three localities in Cambodia (in 2015), and others in Bangladesh (in 2015) [26, 32]. These specimens were acquired using standard techniques of walking through suitable habitat, such as humid forests and forest edges, collecting leeches from the ground or as they attached to the investigators’ clothing. Leeches found feeding on investigators were excluded. Collected leeches were relaxed and fixed in either 95% ethanol or in RNAlater (Ambion). Whole body tissue was sampled just anterior of the caudal sucker and posterior to the mid-body (~ 2.5 mm maximum for larger specimens) for proteolytic digestion and whole-genomic DNA extraction. This area was chosen for extraction as it includes the posterior-most portion of the digestive tract where any residual blood meal is most likely to remain; however, it also includes all of the surrounding leech tissue. Of equal importance, taxonomically informative characters (e.g., the number of caudal sucker rays and the reproductive apparatus) are not damaged by this tissue removal strategy. Unlike the next-gen approach, no regard was given as to the presence of a visible blood meal, and the entire body portion was subject to digestion using a Qiagen DNeasy Blood and Tissue kit.

Amplification reactions (including those for negative controls, of which there were at least 2 per 96-well plate) used 0.5 μL of each 1 μM tetrapod-specific 12Sa (CTGGGATTAGATACCCCACTAT) and 12So (GTCGATTATAGGACAGGTTCCTCTA) primers [33] or trypanosome-specific primers 18S-1f (ACCGWTTCGGCTTTTGTTGG) 18S-4r (CCCCCTGAGACTGTAACCT) targeting the nuclear 18S rRNA gene [34], combined with 23 μL of water, 1 μL of DNA template, and GE illustra PuReTaq Ready-To-Go PCR beads. The thermocycler protocol used in the summer of 2017 included an initial denaturation at 95°C for 3 minutes, followed by 30 cycles at 95°C for 1 minute, 55°C for 30 seconds, and 72°C for 1 minute, followed by a final extension at 72°C for 6 minutes and 40 seconds. Amplification products (including negative controls) were cleaned using Agencourt AMPure XP (Beckman Coulter), cycle sequenced with BigDye (Applied Biosystems), and ethanol-precipitated prior to electrophoresis on an ABI 3730xl (Applied Biosystems). Resulting sequences had primers trimmed, were reconciled, and were edited for quality in Geneious version 6.1.8 (Biomatters).

### Next-generation DNA sequencing

In contrast to the foregoing, leeches subjected to next-generation DNA sequencing in the summer of 2013, were from legacy collections not originally intended for iDNA evaluation. Those included *Chtonobdella fallax* collected from Toliary State Madagascar in 2002, *Haemadipsa interrupta* from the Commonwealth National Forest and from Tasik Kenyir Malaysia in 2007, and *Chtonobdella tanae* from Queensland Australia collected in 2006 [35, 36]. Leeches were identified by morphological characteristics as well as by phylogenetic association with other leeches on the basis of cytochrome *c* oxidase subunits 1 and 3 (*cox1*, *cox3*) in addition to nuclear 18S and 28S ribosomal RNA genes. In order to maximize the probability of acquiring non-leech iDNA, and unlike the conventionally sequenced samples (above), ethanol-preserved leeches were bisected posteriorly to reveal residual blood in the posterior gastric post-ceca while leaving the median male and female reproductive apparatus intact for future studies. Residual blood meals were removed with sterile forceps; intestinal tissue was avoided entirely and digestive epithelial tissues were removed from hardened blood meals. This minimized contamination by leech genomic DNA, allowing for higher success rates of amplification and the ability to isolate symbiotic bacteria (see below). DNA was isolated using the methods detailed above. Isolates of blood meals of the same species from the same localities were pooled to increase DNA input for sequencing.

In addition to whole genomic shotgun DNA library preparations from the iDNA isolates, the *Chtonobdella tanae* isolate also served as template for amplification with bacterial-specific 16S rDNA primers [37, 38], trypanosome-specific 18S rDNA primers [39], and vertebrate specific mitochondrial 12S rDNA primers [27, 31]. Amplification reactions were pooled and processed into a MID-adapter-labelled Lib-L emPCR library that was distinct from the fragmented whole-genomic DNA libraries. Multiplexed emPCR reactions were proportioned for bead recovery in a manner that ~15% would represent libraries constructed from PCR amplifications (5% each of bacterial 16S, trypanosome 18S and vertebrate 12S) and in which 85% would represent whole genomic iDNA isolates (after nebulization and fragment-end repair of the latter). Emulsion-based clonal amplification, bead washes, bead recovery, DNA library bead enrichment, and sequence primer annealing were carried out using the 454 GS Junior Titanium emPCR (Lib-L) protocols as outlined in the *emPCR Amplification Method Manual* (*Lib-L*)(v. April 2011). Enriched beads were prepared for sequencing on a 454 GS Junior PicoTiterPlate Device using the 454 GS Junior Titanium Sequencing Kit following manufacturer’s protocols as outlined in the *Sequencing Method Manual* (v. November 2011). Single-end massively parallel pyrosequencing was carried out in multiplex on a 454 GS Junior at the Sackler Institute for Comparative Genomics (American Museum of Natural History).

Data were demultiplexed and passed through five standard quality filters (Dot, Mixed, Signal Intensity, Primer and TrimBack Valley) using native 454 GS Junior software. Thereafter, sff_extract (http://bioinf.comav.upv.es/sff_extract/index.html) was used to create.fasta, fasta.qual,.fastq, and.xml files. In addition, sff_extract clipped key/adaptor sequences and removed low quality reads (i.e., any base listed in lower case). Sequence quality was visualized using FastQC v 0.10.1 (http://www.bioinformatics.babraham.ac.uk/projects/fastqc). The FASTX Toolkit v 0.0.13 was used to further remove low quality regions yielding sites with Phred quality scores ≥25.

### Sequence characterizations

Conventionally sequenced tetrapod 12S rDNA and trypanosome 18S rDNA reads were identified using blastn to NCBI’s nucleotide collection (nr/nt) and discarding all results that failed to match the candidate locus. In order to prepare candidate read sets for assembly, next-generation DNA sequence reads first were compared against a local database of eight vertebrate whole mitochondrial genomes covering amphibians, birds, squamates and mammals, the 10 chromosomes and the mitochondrial genome of *Trypanosoma brucei*, all bacterial and archaeal genomes, and the *Helobdella robusta* leech genome (BioProject: PRJNA175704). Only reads scoring better than 1e^-10^ and scoring at least two orders of magnitude better than matches to *H*. *robusta* (to avoid leech contamination) were retained and assembled with CodonCode Aligner (v8.0.2).

Following previously defined protocols [26, 32] final identifications were made based on the lowest e-value while noting whether or not the best match was at least a 3% better match than the taxonomically next-best e-value returned (assumed to be the case if the first 100 matches were the same species). Wherein several taxa had identical e-values, an identification was made only to the lowest rank at which there was discernment (i.e., genus or family).

For bacterial 16S rDNA from *C*. *tanae*, phylogenetic determination of the relative placement of sequence reads in the Rikenellaceae used previously published data [39] for aquatic leech crop-associated microbes (aligned length = 2214). For trypanosomes, we incorporated relevant 18S rDNA available through NCBI based on the closest matching BLAST hits and an additional range of trypanosome sequences rooted with the aquatic *Trypanosoma grayi* group (alignment length = 2513). In each case, sequences were aligned with MUSCLE (v3.8) [40] and analyzed with PhyML (v3.0) [41] with a GTR+I+G model through www.phylogeny.fr, a free, simple to use web service dedicated to reconstructing and analysing phylogenetic relationships between molecular sequences.

## Results

**Fig 1:**
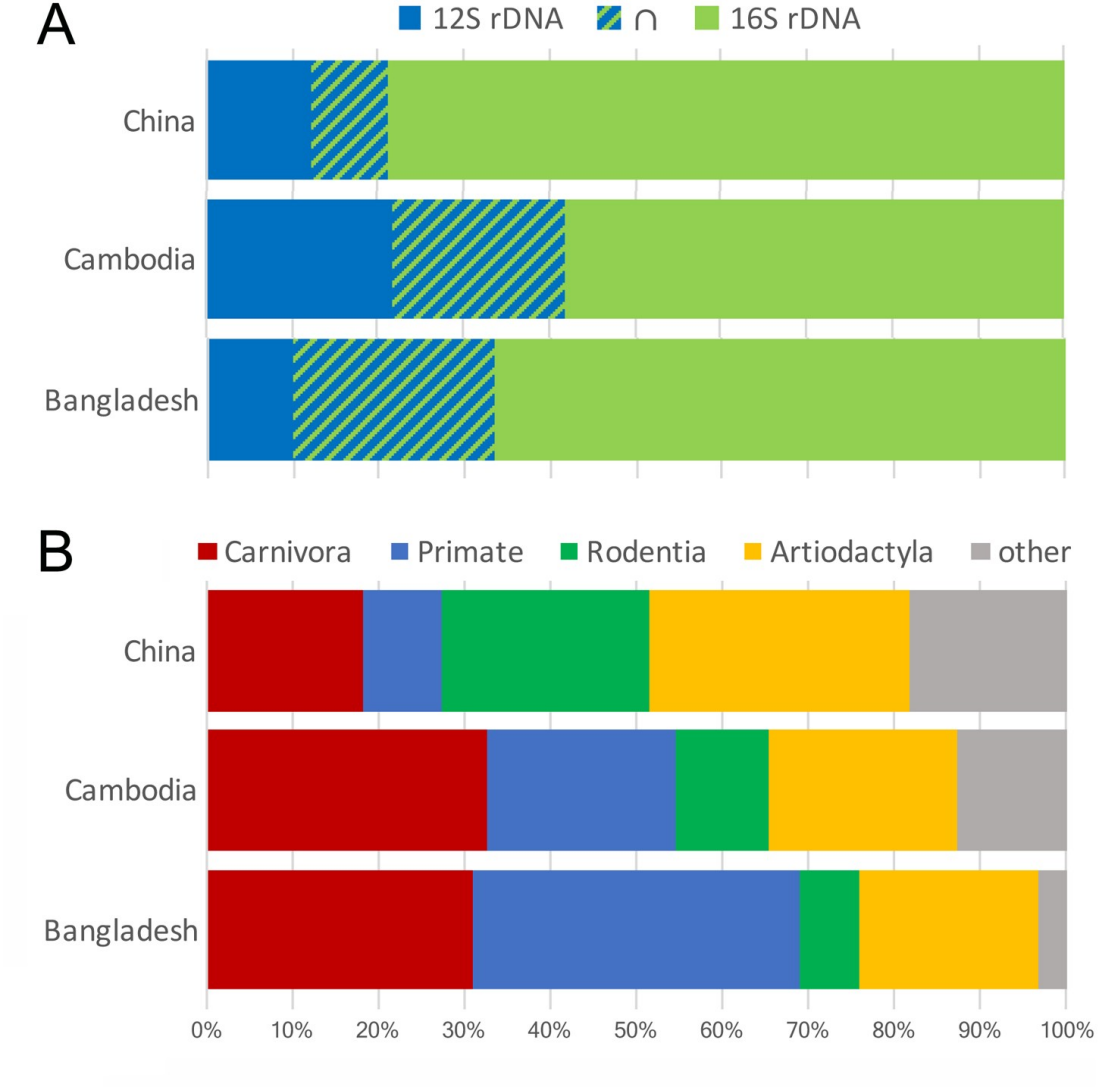
Representation of high-scoring conventional sequence reads in terrestrial haemadipsid leech iDNA. A) proportional to locus in which leeches that amplified for both loci from China, Cambodia and Bangladesh represented 9%, 20%, and 24% of samples respectively; B) the union set proportional to tetrapod orders excluding humans and domestic animals. See S1 Table.

Among the 761 individual leeches examined (S1 Table) with conventional sequencing methods, 437 did not yield an amplification product either for 16S rDNA or for 12S rDNA. Excluding highest-scoring sequence matches to humans, cattle, water buffalo, pig, chicken and house-rats, there were 39 confirmed high-scoring (better than 1e^-20^) sequences from 12S rDNA. Twenty-one (54%) of those samples also previously yielded matches to non-human and non-domesticated animals on the basis of 16S rDNA. Altogether, 12S rDNA and 16S rDNA data combined were dominate by carnivores, primates and artiodactylids (Fig 1B). The proportion of leeches from which DNA was extracted without regard to the visual presence or absence of bloodmeals that yielded sequence information relative to animals of interest (15%) well-exceeds the 5% of leeches observed with visible blood meals in legacy collections examined here with next-gen sequencing (below). Average sequence identity to high scoring matches in nr/nt was 97.7% for 12S rDNA and 95.9% for 16S rDNA.

Among those isolates conventionally sequenced for 12S rDNA, tetrapod species identified came from three classes (S1 Table): Mammalia, represented by six orders (Artiodactyla, Carnivora, Chiroptera, Eulipotyphla, Primates, Rodentia); Aves, represented by two orders (Galliformes; Strigiformes); and Amphibia, represented by one order (Anura). Among those isolates previously conventionally sequenced for 16S rDNA, tetrapod species identified came from two classes: Mammalia, represented by five orders (Artiodactyla, Carnivora, Chiroptera, Primates, Rodentia); and Aves, represented by one order (Galliformes). In Bangladesh, most iDNA isolates were primate (38%, esp. macaque), carnivore (31%, esp. civets) or ungulate (21%, esp. muntjak). Similarly, across Cambodia, most isolates were carnivore (33%, esp. civet), primate (22%, esp. macaque) or ungulate (22%, esp. red deer and mouse deer). Isolates from China were predominantly ungulate (30%, esp. muntjak and red deer), rodent (24%, esp. white bellied rat) or carnivore (18%, esp. civet).

In a manner not apparent with the mammal specific 16S rDNA locus, individual sample amplifications and conventional sequencing of the 12S rDNA locus provided results that included amphibians like a microhylid from Cambodia (e-value = 1e^-14^, 98% identity) and a species of *Fejervarya* from China (e-value = 6e^-52^, 99% identity). While it also found matches to birds, none of these presented convincing scores both for e-value and sequence identity. Rodents also were better represented among 12S rDNA sequences (24%) than among 16S rDNA data (2%) from China, Bangladesh and Cambodia combined. In the case of both loci, many species were recovered only once: a Northern treeshrew (*Tupaia belangeri*, e-value = 8e^-114^, 96% identity) from Bangladesh, each of two squirrels (*Menetes berdmorei*, e-value = 5e^-29^, 96% identity; *Proscirillus murinus*, e-value = 8e^-22^, 100% identity), a gaur (*Bos gaurus*, e-value = 5e^-133^, 99% identity), and a roundleaf bat (*Hipposideros bicolor*, e-value = 4e^-73^, 93% identity) from Cambodia, as well as one squirrel (*Dremomys rufigenis*, e-value = 3e^-106^, 93% identity), a mole (*Scapanulus oweni*, e-value = 2e^-20^, 97% identity), and a silver pheasant (*Lophura nycthemera*, evalue = 1e^-125^, 96% identity) from China.

It was apparent that method of field preservation influenced amplification success. All samples from China and Bangladesh had been fixed in RNAlater; samples from Cambodia had been preserved in either RNAlater or in 95% ethanol. Of the 55 samples representing target wild taxa, 39 had been fixed in RNAlater and 16 in 95% ethanol, and of the 23 Cambodian samples providing vertebrate 12S rDNA sequences, only 2 had been preserved in ethanol. Regardless, amplification and sequencing of 12S rDNA was confirmatory of prior 16S rDNA results in 7 samples from Bangladesh, 11 samples from Cambodia, and 3 samples from China. It also provided additional data for 3 samples from Bangladesh, 12 samples from Cambodia, and 4 samples from China, none of which previously yielded data for 16S rDNA.

The vertebrate 12S locus could not discriminate among species of macaque from Bangladesh or Cambodia (see S1 Table), whereas the 16S locus was able to distinguish *Macaca mulatta* and *Macaca sinensis* with sequence identities of 97% or higher. Conversely, scores of 87% to 95% from 16S rDNA suggesting African civets (*Civettictis civetta*) in Bangladesh which were properly identified as the Large Indian civet (*Vivera zibetha*) at 100% sequence identity with 12S rDNA. Nonetheless, there were also disagreements between loci. A herpestid from Bangladesh was determined by 12S rDNA to be *Herpestes javanicus* (evalue = 1e^-43^, 99% identity), not an African *Crossarchus obscurus* (evalue = 1e^-107^, 93% identity) as suggested by 16S rDNA. In contrast, a rodent from Cambodia was more plausibly identified with 16S rDNA as *Niviventer excelsior* (evalue = 4e^-41^, 92% identity) as opposed to being an Amazonian *Oecomys auyantepui* (evalue = 1e^-24^, 95% identity) as suggested by 12S rDNA. From China, porcupine iDNA from one sample was either *Athurus macrourus* (evalue = 1e^-30^, 100% identity on 12S rDNA) or *Hystrix indica* (evalue = 1e^-105^, 93% identity on 16S rDNA), both of which are geographically plausible.

Genomic iDNA next-gen sequences from the Australian leech *C*. *tanae* yielded significant scores against a pademelon (*Thylogale billardierii*: tRNA-Leu+nd1, evalue = 3e^-142^), humans (*Homo sapiens*: 12S, evalue 0.0; cox3, evalue = 0.0; cytb+tRNA-Thr, evalue = 0.0), and a domestic dog (*Canis lupus familiaris*: cox2, evalue = 0.0; nd4, evalue = 2e^-159^; nd5, evalue = 0.0). Reads from the Malaysian leech *H*. *interrupta* matched those of a mouse deer (*Tragulus javanicus*: tRNA-Phe, 6e^-101^; tRNA-Trp+tRNA-Ala+tRNA-Asn+tRNA-Cys+tRNA-Tyr+cox1, evalue = 0.0; cox1, evalue = 0.0; cox2, evalue = 0.0; nd3+tRNA-Arg+nd4L, evalue = 0.0; nd2 evalue = 0.0; nd4 evalue = 0.0; nd4+tRNA-His+tRNA-Ser+tRNA-Leu+nd5 evalue = 0.0) with an overall 97.9% sequence identity. Those from the Madagascan *C*. *fallax* returned significant matches for the lesser hedgehog tenrec (*Echinops telfairi*: cox1, evalue = 1e^-123^), for the ring-tailed mongoose (*Galidia elegans*: nd2, evalue = 0.0; cytB, evalue = 0.0; tRNA-Thr+tRNA-Pro+control region, evalue = 0.0) with an overall 98.8% sequence identity, and in particular for the narrow-striped mongoose (*Mungotictis decemlineata*: nd5, evalue = 0.0; nd4, evalue = 0.0; atp6+cox3, evalue = 0.0; tRNA-Phe+12S evalue = 1e^-159^; nd1, evalue = 2e^-167^; cox1, evalue = 0.0; cox1+tRNA-Ser+tRNA-Asp+cox2, evalue = 0.0; nd5+nd6, evalue = 0.0) with an overall 91.5% sequence identity.

**Fig 2:**
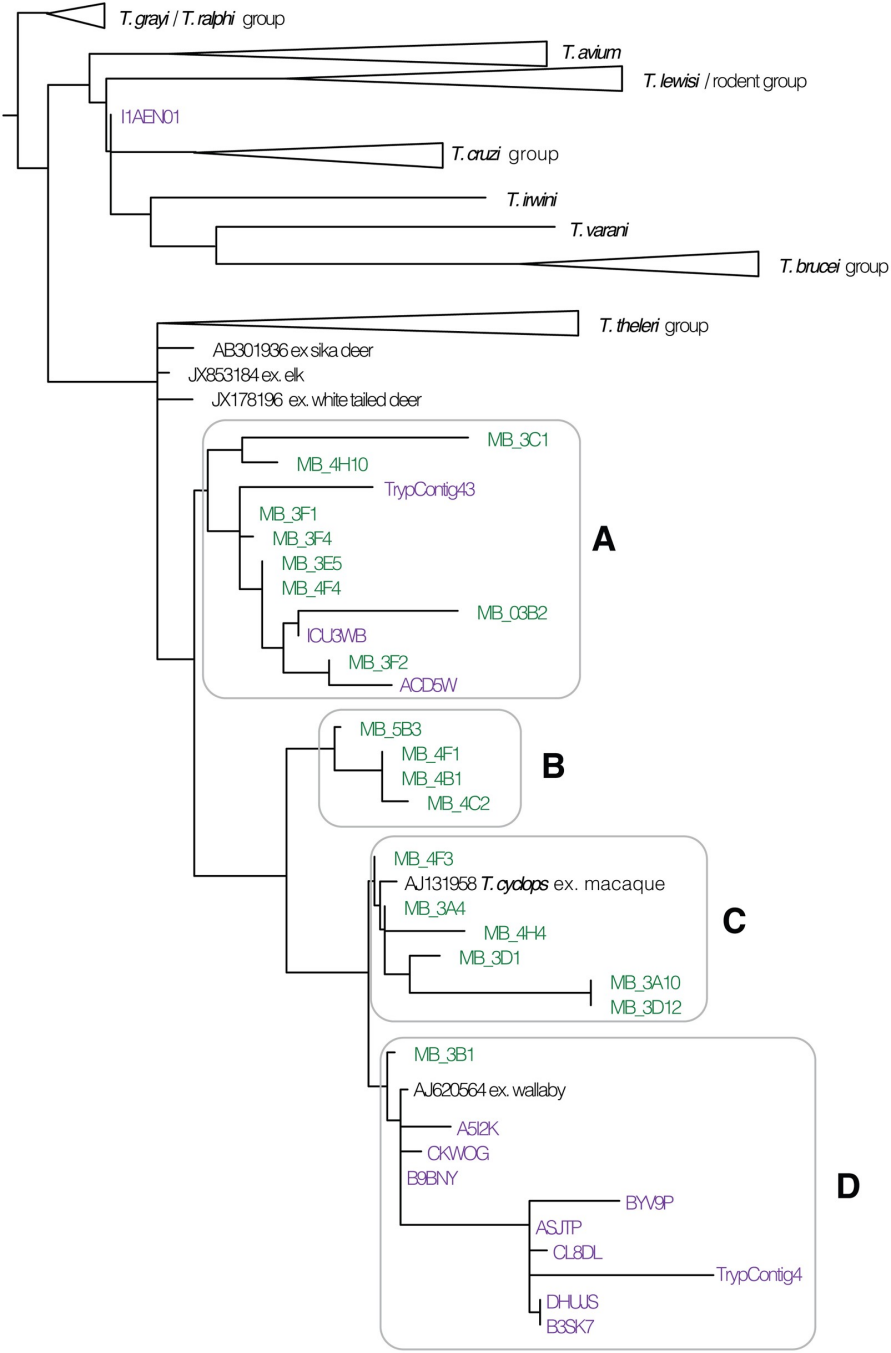
Optimal tree for maximum likelihood analysis of tetrapod-infecting trypanosome 18S rDNA data. Recovered from conventional recently sampled (green; Southern Asia) and next-generation legacy sample (violet; Australia) sequencing of leech gut iDNA in the context of comparable data in public databases. Branch lengths are proportional to number of substitutions per site. Terminal triangles represent clades of multiple samples.

Thirteen isolates amplified and sequenced conventionally yielded 18S rDNA reads for trypanosomes. One resulted from a specimen of *Haemadipsa rjukjuana* (specimen 5B3) from Goaligong, China, that had human 16S rDNA in its gut. The remaining 12 were specimens of *Haemadipsa ornata* from Bangladesh. Two evidenced muntjak iDNA (3C1, 4H10), three had cow iDNA (3E5, 3B2, 4C2), two had pig iDNA (4F1, 4C2), one had macaque iDNA (3A4), and one evidenced mongoose iDNA (4H4). Next-generation sequence reads resulting from amplifications of trypanosome 18S rDNA only yielded best matches from nr/nt for pooled samples of *C*. *tanae*: 72 reads assembling into 2 contigs and 11 singletons. Phylogenetic analysis of all of the foregoing conventionally and next-generation sequenced 18S rDNA reads in combination with those of putatively related species of *Trypanosoma* revealed four principal clades (Fig 2), all of which were relatively closely related to *Trypanosoma theileri* and *Trypanosoma cyclops* (clade C in Fig 2). Conventional reads from leeches that had fed on a macaque (3A4) grouped with *Trypanosoma cyclops* (clade C in Fig 2) previously only known from macaques, as did isolate 4H4 that had fed on a mongoose. Conventional reads from leeches that had fed on muntjak (3C1 and 4H10) grouped together in a clade (clade A in Fig 2) with others from leeches that had cow iDNA and with reads obtained from next generation sequencing of *C*. *tanae*. However, the clade with the most reads obtained from next generation sequencing of *C*. *tanae* grouped with an unnamed trypanosome isolate previously known from a wallaby (clade D in Fig 2).

**Fig 3:**
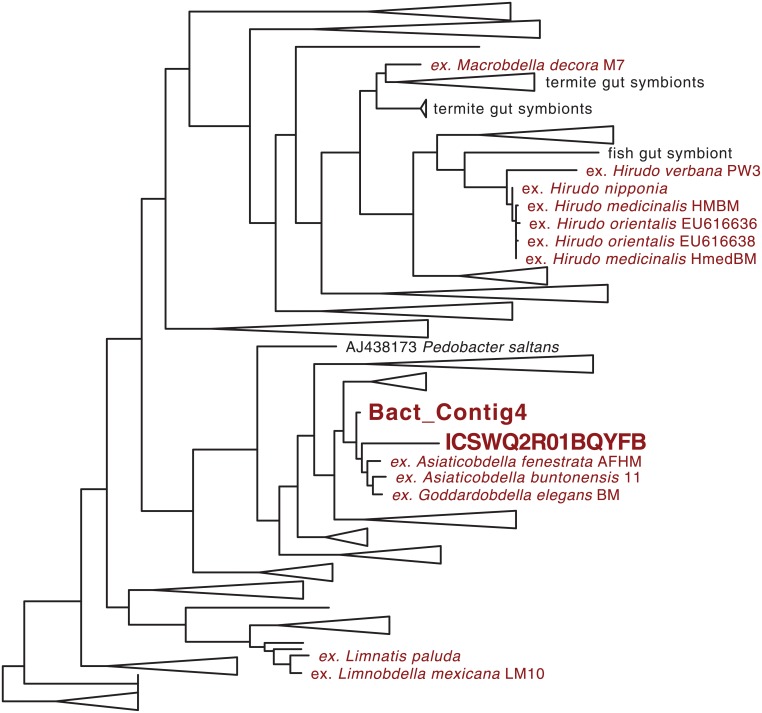
Optimal tree for maximum likelihood analysis of Rikenellaceae 16S rDNA. including those previously obtained in crop symbiosis from various leech species (red) as well as those obtained here from the gut of *Chtonobdella tanae* (bold red). Branch lengths are proportional to number of substitutions per site. Terminal triangles represent clades of multiple samples.

Next-generation sequence reads resulting from amplifications of tetrapod 12S rDNA from *C*. *tanae* numbered 77 that assembled into 9 contigs, of which 7 were human and one was a dog. Next-generation sequence reads resulting from amplifications of bacterial 16S rDNA from *C*. *tanae* numbered 143 that assembled into 7 contigs, leaving 9 singletons, and yielding best scoring matches (evalue < 2e^-21^) to cultured and uncultured microbial taxa: Corynebacteriales, Rikenellaceae, beta-proteobacteria, alpha-proteobacteria, Prevotellaceae, and Firmicutes. The contig representing the largest proportion (45%) of reads from amplifications, and an additional single read left out of all contigs, were both closely related to other Rikenellaceae previously recovered from the digestive tract of Australian and African aquatic leeches in the family Hirudindae (Fig 3).

In terms of best scoring genome-wide matches by genomic iDNA contigs to available microbial sequences in nr/nt, the most frequently recovered (Fig 3) were species of *Mesorhizobium*, *Aminobacter*, and other Rhizobiales (44%, median evalue = 2e^-113^), as well as *Delftia* species and other Burkholderiales (23%, median evalue = 4e^-26^). The remainder was distributed across alpha-proteobacteria (8%), beta-proteobacteria (8%), gamma-proteobacteria (8%), Actinobacteria (4%), and nitrogen fixing Rhodospirillaceae (4%).

## Discussion

Our findings reinforce the notion that amplifiable DNA in legacy museum specimens can reveal aspects of an organisms’ biology and ecological interactions with the surrounding community [3]. Specifically, these terrestrial leech specimens were able to provide information regarding geographically co-occurring tetrapods and their blood parasites as well as for the leeches themselves and their associated microbiomes. Significantly, this study provides further insight into best practices regarding standardizing iDNA protocols [24, 26, 27, 32]. Unlike studies leveraging blood-feeding dipterans [23] visible blood meals in the crop of leeches appear to be unnecessary for the success of iDNA biodiversity surveys. And while new studies might consider the superiority of RNAlater over ethanol preservation in the field for de novo comprehensive surveys, it is clear that greater-than decade-old ethanol-preserved samples decade-old retain sufficient mitochondrial DNA to be of complementary value.

As inferred elsewhere [23] amplification and sequencing of both 12S and 16S loci is preferable to a single-locus iDNA approach however, study designs for indirect assessments of biodiversity, be that through iDNA surveys of leeches or eDNA from water sources [42], still need to account for the relative unevenness of coverage of reference databases. From the next-generation shotgun genomic sequencing approach there was a precise lack of overlap among mitochondrial loci obtained and identified as Malagasy mongoose species: *Galidia elagans* versus *Mungotictis decemlineata*. Where loci were represented for *Galidia elegans* in public reference databases, those received strong (>98%) matches from next-gen data; and where those loci did not exist for *G*. *elegans*, next-gen data matched loci in the mitochondrial genome of *M*. *decemlineata* exceedingly well (>91%). Thus, we conclude that the leech had fed on a *G*. *elegans* not on a *M*. *decemlineata* notwithstanding a larger number of sequences matching a larger number of loci for *M*. *decemlineata*, because the reference database includes fewer loci for the former. Had we only sequenced a single target locus, including the *cox*1 barcoding locus, we would have readily concluded otherwise. Nor for that matter does a lower e-value on one locus necessarily imply higher confidence in taxonomic identification relative to a worse e-value on a different locus especially without reference to how close a match the next-best taxon might be. Without thorough knowledge of the structure and completeness of the reference databases against which environmental samples are to be compared, blithe and uncritical acceptance of high-scoring matches is bound to be misleading in ways that are worse than merely uninformative. This should also discourage any clustering of sequencing reads into MOTUs prior to taxonomic assignment [43] with an arbitrarily chosen level of similarity. The mean sequence identity recovered against *Tragulus javanicus* for more than half a dozen loci exceeded the 97% identity measure often used for clustering MOTUs regardless of the fact that this species is not known to exist in Malaysia. Yet scores against available data for the Malaysian *Tragulus kanchil* were worse. We take this to be indicative of a different Malaysian taxon (e.g., *Tragulus napu*) for which no comparable data exist. The possibility of acquiring sequence information for undescribed taxa should not be ignored [44].

Any source of direct or indirect data on forest biodiversity entails unique biases (Fig 1). Leech iDNA, while more adept at recovering smaller ground-dwelling tetrapods than camera traps [32], are presumed to be insensitive to arboreal-dwelling species (e.g, lemurs) where unlike dipterans [23] leeches spend little time. Nonetheless, it is apparent from the combined 12S and 16S data that exploiting the gut contents of tropical terrestrial haemadipsid leeches has the potential to sample non-mammalian and mammalian diversity alike. Next-generation amplicon studies employing additional primer sets in combination that are exclusive and specific to amphibians, squamates or birds might yet recover more diversity than was apparent here [18].

Natural history collections serve not only as fundamental libraries of spatiotemporally dynamic patterns of biodiversity, for example of vertebrate taxa in regions exposed to climate change, but also serve as an invaluable resource relating to their associated parasites, pathogens and microbiotas [45]. In many cases, first-appearances, historical distributions, and range-shifts of parasitic helminths and protists, have only be recovered from tissues and related information originally collected with the intent of the host alone [46, 47]. Understanding parasite diversity has itself been driven by retrospective assessments of existing collections of their hosts [48, 49, 50]. Trypanosomes are especially well retained in and amplifiable from bloodfeeding vectors like tsetse flies [51]. Fig 2, based on isolates from leeches, suggests that there is a greater diversity yet to be characterized and taxonomically described from terrestrial Australian tetrapods, particularly as it concerns clades A and B. Similarly, amplifiable trypanosome DNA was recovered from 25 of 44 terrestrial leeches sampled in Australia and New Guinea [52], much like finding trypanosomes in the digestive tracts of leeches up to 44 days after feeding [53]. None of this, however, should be taken as evidence of leeches playing a functional role in vectoring trypanosomes among terrestrial tetrapods. Regrettably, ozobranchid leeches have been implicated as a vector of marine turtle herpesvirus [54, 55] in light of finding viral DNA in their gut contents yet without any positive evidence of transmission by those leeches. Likewise, trypanosomatids are demonstrably viable even *in vitro* with minimal nutritional provision [56, 57]. As such their presence in the crop of any invertebrate should not be taken as evidence of the latter’s status as a viable infection vector. Nevertheless, whatever diversity can be characterized from iDNA is meaningfully representative of the hematozoa resident in geographically coincident vertebrate hosts.

Outside of serious infection from resident gut flora of aquatic leeches [58, 59, 60] there has been no prior evaluation of the microbiome of terrestrial haemadipsid leeches notwithstanding their frequent ectoparasitism of humans [61, 62, 63]. Metagenomic data obtained here for pooled samples of *C*. *tanae* from Australia suggest that species of *Aeromonas* uniformly found in the crop of “medicinal” leeches [64, 65, 66, 67] and implicated in human infections are not well-represented in the crop of terrestrial leeches. Other stereotypical leech gut related symbionts from Rikenellaceae [39, 68, 69] are present in terrestrial leeches, with isolates grouping biogeographically (Fig 3). Lineages obtained here from *C*. *tanae* group with other Gondwanan (African and Australian) taxa. There is also an apparent unambiguous symbiotic association for Rhizobiaceae with heamadipsid leech crop contents in a manner that is strikingly similar to what was discovered for obligate intracellular mycetome endosymbionts of freshwater leech species in the *genus Placobdella* [28, 38]. Presently, unlike the intracellular bacterial symbionts of *Placobdella* species there does not appear to be a tissue-specific relationship for these microbes that otherwise are best known for symbioses in leguminous plants. Further careful morphological work will be required to assess whether haemadipsid crop-associated “Lambertion organs” [70] are the location of these bacteria and whether, like the esophageal bacteria of the genus *Placobdella*, the bacteria retain all of the functional iron-dependent nitrogen-fixing genetic loci.

## Supporting information

S1 Table Highest-scoring sequence matches against NCBI nr/nt for conventional amplification and sequencing of 12S rDNA and 16S rDNA for iDNA obtained from individual leeches. These data exclude those leeches for which the best match was human or domesticated animal, and include only those for which the percent sequence identity was approximately 90% or better and at least 3% better than the next best taxonomically different match.
(XLSX)
